# Differences in intrinsic aerobic capacity alters sensitivity to ischemia-reperfusion injury but not cardioprotective capacity by ischemic preconditioning in rats

**DOI:** 10.1371/journal.pone.0240866

**Published:** 2020-10-27

**Authors:** Marie Vognstoft Hjortbak, Thomas Skjærlund Grønnebæk, Nichlas Riise Jespersen, Thomas Ravn Lassen, Jacob Marthinsen Seefeldt, Pernille Tilma Tonnesen, Rebekka Vibjerg Jensen, Lauren Gerard Koch, Steven L. Britton, Michael Pedersen, Niels Jessen, Hans Erik Bøtker

**Affiliations:** 1 Department of Cardiology, Aarhus University Hospital, Aarhus, Denmark; 2 Department of Clinical Medicine, Aarhus University, Aarhus, Denmark; 3 Department of Public Health, Aarhus University, Aarhus, Denmark; 4 Department of Physiology and Pharmacology, The University of Toledo College of Medicine and Life Sciences, Toledo, Ohio, United States of America; 5 Department of Anesthesiology, University of Michigan, Ann Arbor, Michigan, United States of America; 6 Department of Molecular and Integrative Physiology, University of Michigan, Ann Arbor, Michigan, United States of America; 7 Steno Diabetes Center Aarhus, Aahus University Hospital, Aarhus, Denmark; 8 Department of Clinical Pharmacology, Aarhus University Hospital, Aarhus, Denmark; 9 Department of Biomedicine, Aarhus University, Aarhus, Denmark; Indiana University School of Medicine, UNITED STATES

## Abstract

**Introduction:**

Aerobic capacity is a strong predictor of cardiovascular mortality. Whether aerobic capacity influences myocardial ischemia and reperfusion (IR) injury is unknown.

**Purpose:**

To investigate the impact of intrinsic differences in aerobic capacity and the cardioprotective potential on IR injury.

**Methods:**

We studied hearts from rats developed by selective breeding for high (HCR) or low (LCR) capacity for treadmill running. The rats were randomized to: (1) control, (2) local ischemic preconditioning (IPC) or (3) remote ischemic preconditioning (RIC) followed by 30 minutes of ischemia and 120 minutes of reperfusion in an isolated perfused heart model. The primary endpoint was infarct size. Secondary endpoints included uptake of labelled glucose, content of selected mitochondrial proteins in skeletal and cardiac muscle, and activation of AMP-activated kinase (AMPK).

**Results:**

At baseline, running distance was 203±7 m in LCR vs 1905±51 m in HCR rats (p<0.01). Infarct size was significantly lower in LCR than in HCR controls (49±5% vs 68±5%, p = 0.04). IPC reduced infarct size by 47% in LCR (p<0.01) and by 31% in HCR rats (p = 0.01). RIC did not modulate infarct size (LCR: 52±5, p>0.99; HCR: 69±6%, p>0.99, respectively). Phosphorylaion of AMPK did not differ between LCR and HCR controls. IPC did not modulate cardiac phosphorylation of AMPK. Glucose uptake during reperfusion was similar in LCR and HCR rats. IPC increased glucose uptake during reperfusion in LCR animals (p = 0.02). Mitochondrial protein content in skeletal muscle was lower in LCR than in HCR (0.77±0.10 arbitrary units (AU) vs 1.09±0.07 AU, p = 0.02), but not in cardiac muscle.

**Conclusion:**

Aerobic capacity is associated with altered myocardial sensitivity to IR injury, but the cardioprotective effect of IPC is not. Glucose uptake, AMPK activation immediately prior to ischemia and basal mitochondrial protein content in the heart seem to be of minor importance as underlying mechanisms for the cardioprotective effects.

## Introduction

Aerobic capacity is a strong predictor of cardiovascular mortality [1–3]. Regular exercise increases aerobic capacity and favourably adjusts many known cardiovascular risk factors of coronary artery disease [4]. In contrast, sedentary lifestyle and low aerobic capacity may increase the risk of coronary artery disease by increasing the same risk factors [3,5], and even cause obesity and metabolic syndrome, which may further complicate the disease profile. The level of daily physical activity and exercise can modulate aerobic capacity, but other factors such as genetic background also contribute [6]. Aerobic capacity may not only influence cardiovascular mortality by modulating risk factors but also by influencing susceptibility to ischemia reperfusion (IR) injury [7].

A common method to induce cardioprotection is ischemic conditioning, which consists of brief non-lethal episodes of ischemia and reperfusion prior to a lethal period of ischemia [8,9]. The concept of ischemic conditioning can be applied locally on the heart as ischemic preconditioning (IPC) or as remote ischemic conditioning (RIC), which is a more clinically applicable approach [10]. IPC and RIC have shown substantial cardioprotective effect in preclinical studies, but translating the favourable effect to significant clinical benefits remains challenging [11,12]. Most preclinical studies have been performed in healthy young animals that do not represent the complex nature of a clinical population suffering from acute myocardial infarction [13]. Differences in aerobic capacity may reflect parts of the variation in a clinical setting, and exercise has been shown to interact and even share mechanisms with conditioning strategies [14].

The aim of the present study was to investigate the impact of aerobic capacity on IR injury, and to study the influence of aerobic capacity on the cardioprotective efficacy of IPC and RIC in an experimental model using hearts from rats with high or low running capacity.

## Methods

### Animals

We studied male rats selectively bred to be either high capacity (HCR) or low capacity (LCR) runners. 48 HCR rats and 50 LCR rats were included in total. The development of the rat model of HCR and LCR rats has been comprehensively described previously [15,16]. The rat model displays two very distinct phenotypes which, in addition to large differences in running capacity, present substantial differences in both aerobic capacity and also other characteristics such as body composition, blood levels of lipids and glucose [15,17,18]. The rats used in the current study were from the 26th and 40th breeding generation, and had a mean age of 8 months. All animals were housed at a constant temperature of 23°C with a 12-hour light-dark cycle and allowed unlimited access to standard chow and water. All animals were tested for running capacity according to standard procedure in the breeding programme [17].

All animal experiments were carried out in agreement with the Danish law for animal research (Act. No. 1306 of 23/11/2007, Danish Ministry of Justice) and the guidelines from *Guide for the Care and Use of Laboratory Animals published* by the US National Institutes of Health (NIH Publication No. 85–23, revised 1996). The Danish Animal Experimental Inspectorate approved the experimental work (authorization id. 2012-15-2934-00623 and 2018-15-0201-01446).

### Experimental protocols

**Fig 1:**
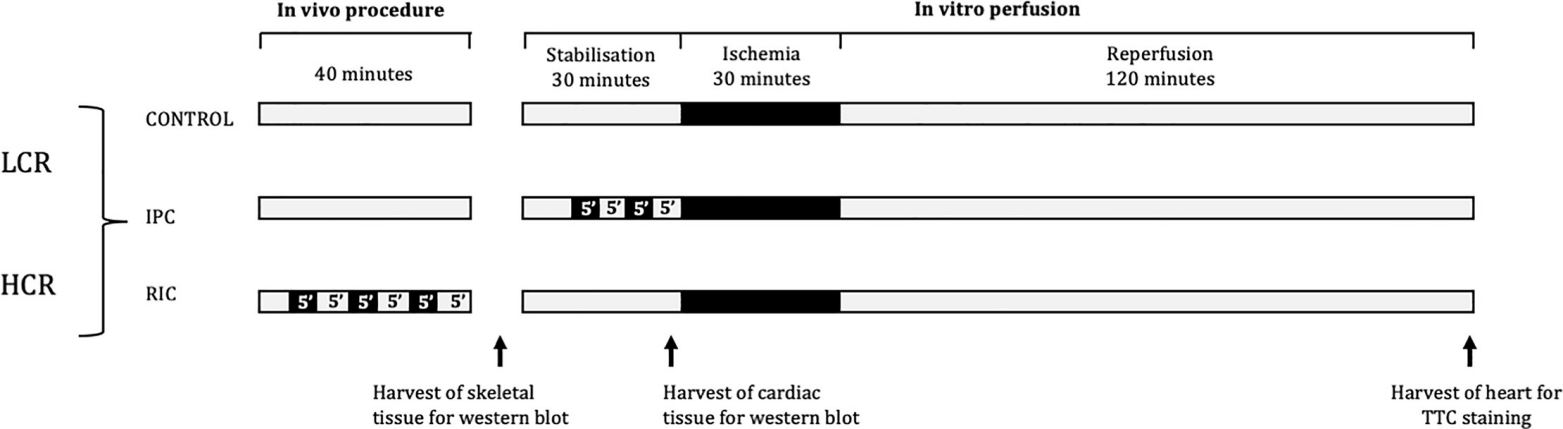
Study design. Study protocols used. LCR: Low capacity runners, HCR: High capacity runners, IPC: Local ischemic preconditioning, RIC: Remote ischemic preconditioning. The whole protocol was performed at 37±0.5°C.

All animals were subjected to 40 minutes of *in vivo* procedure, followed by isolation and *in vitro* perfusion of the heart in the Langendorff model. Hearts were then subjected to 30 minutes stabilisation, 30 minutes global no-flow ischemia and 120 minutes reperfusion. HCR and LCR rats were randomized to either: group I. Control, group II. IPC, or group III. RIC (Fig 1). Control and IPC groups received no intervention during the *in vivo* procedure. IPC was applied in the isolated perfused hearts by two cycles of 5 minutes of global no-flow ischemia and 5 minutes of reperfusion prior to index ischemia. RIC was applied *in vivo* by three cycles of 5 minutes limb ischemia and 5 minutes reperfusion. Limb ischemia was achieved with a tourniquet around the right hind leg. Ischemia was verified by paling of the foot followed by hyperemia during reperfusion.

Skeletal muscle samples from the tibialis anterior muscle were harvested quickly after isolation of the heart. Cardiac muscle from the left ventricle was harvested in a separate series, after 40 minutes of stabilisation (Fig 1).

### Isolated perfused heart model

Animals were anesthetized with pentobabiturate (100 mg/kg body weight (Skanderborg Pharmacy, Skanderborg, Denmark)), connected to a rodent ventilator (Ugo Basile 7025 rodent ventilator, Comerio, Italy), and ventilated with atmospheric air during the *in vivo* procedure and the isolation of the heart. The isolated perfused heart model was performed according to standard protocol in our laboratory, as previously described [19,20]. The rats underwent laparotomy and thoracotomy, the hearts were rapidly dissected free from the surrounding structures, and a ligature with a tourniquet was placed around the aorta. The animals were heparinized by injection of 1,000 IU/kg heparin bolus through the femoral vein. A cannula was placed in the ascending aorta and retrograde perfusion was established *in situ* with Krebs-Henseleit buffer (11 mM glucose). The hearts were rapidly excised and mounted in a Langendorff apparatus and perfused at a constant pressure of 80 mmHg. The perfusion buffer was equilibrated with 95% O_2_ and 5% CO_2_ to maintain a pH between 7.35–7.45. The temperature was kept constant at 37±0.5°C during the whole perfusion protocol. The left atrial auricle was resected and a balloon-catheter (size 7, Hugo Sachs Electronics, March-Hugstetten, Germany), connected to a pressure transducer, was inserted into the left ventricular cavity for continuous hemodynamic measurements. The balloon volume was adjusted to obtain a left ventricular end-diastolic pressure of 7–10 mmHg. The coronary flow was continuously measured by an in-line flow probe (Hugo Sachs Electronics, March-Hugstetten, Germany). All data were acquired and digitally analysed using a dedicated core software platform (Notocord Hem evolution, Croissy sur Seine, France).

Exclusion criteria were left ventricular developed pressure (LVDP) below 110 mmHg at the end of stabilisation, coronary flow of more than 20 mL/minute or continuous ventricular fibrillation during stabilisation or reperfusion.

### Infarct size

At the end of reperfusion hearts were removed from the perfusion apparatus and immediately frozen at −80°C for a minimum of 20 minutes and subsequently cut into ≈1.5 mm slices. Slices were immersed in 1% 2,3,5-triphenyltetrazolium chloride (Sigma, St Louis, MO, USA) at 37°C and pH 7.4 for 3 minutes rendering vital tissue deep red and infarcted tissue pale. Hearts were stored in 4% formaldehyde (Lillies Solution, VWR–Bie & Berntsen, Herlev, Denmark) for 20–28 hours to enhance the contrast between vital and infarcted tissue. Each heart slice was weighed and scanned on a flatbed scanner (Epson Perfection V600 Photo Scanner, Epson America). The area of left ventricle (LV), which corresponded to the area at risk (AAR), and area of infarction (IS) were assessed manually by observer delineation using computer assisted planimetry (ImageJ 1.46r, Wayne Rasband, National Institutes of Health, USA). Infarct size was expressed as a percentage IS of AAR. Measurements were adjusted to the wet weight of each individual slice. All analyses were performed in a blinded manner.

### Glucose uptake

Myocardial glucose uptake rate was assessed from the rates of ^3^H_2_O production from d-[2-^3^H]-glucose added to the KH buffer [21,22]. The ^3^H_2_O was measured in perfusate samples of 1 mL withdrawn at 9 and 29 minutes of pre-ischemic perfusion, and at 2, 3, 5, 10, 15, 20 and 30 minutes of post-ischemic perfusion. Separation of ^3^H_2_O from d‐[2‐^3^H]‐glucose was performed by anion exchange chromatography on an AG 1-X8 resin column (Bio-Rad Laboratories, Hercules, CA, USA). The ^3^H_2_O was dissolved in 10 mL OptiPhase scintillation solution (Perkin-Elmer, Shelton, CT, USA) and the amount was quantified by β-scintillation counting on a Tricarb^®^ 2900TR liquid scintillation analyser (Packard). The glucose concentration was calculated by dividing the disintegrations per minute by the specific activity of glucose in the perfusate. The results were plotted against time and coronary flow at sample time, and the glucose uptake rate was calculated.

### Western blot analyses

Western blot analyses were performed in two separate series. In the first series, we used cardiac tissue to assess how IPC and RIC affected enzymes involved in substrate metabolism and related the mitochondrial proteins: AMP-activated Kinase (AMPK), Acetyl-CoA Carboxylase (ACC), Glycogen Synthase (GS), Prohibitin-1 (PHB1) and Voltage-dependent anion channel (VDAC). In the second series we used both skeletal and cardiac tissue to assess the content of 3 mitochondrial proteins: Cytochrome c oxidase IX (COX IX), β-hydroxyacyl-CoA dehydrogenase (β-HAD) and citrate synthase (CS).

The Western blot analyses were performed in accordance with standard protocols, as previously described [23,24]. In short, frozen skeletal and cardiac muscle tissue samples were freeze dried, homogenized, and adjusted for equal protein concentration. Equal amounts of protein were separated with SDS-PAGE and electroblotted onto PVDF membranes. Membranes were blocked for 2 hours in a TBST solution with 5% BSA and incubated overnight at 4°C in primary antibodies. The following primary antibodies were purchased from Cell Signaling Technology and utilized as follows: P-AMPK (#2531), AMPK (#2532), P-ACC (#3661), ACC (#3662), p-GS (#3891), GS (#3886), PHB_1_ (#2426), VDAC (#4661), COX IV (#4844, conc. 1:1000 (skeletal muscle) and 1:3000 (cardiac muscle) 5% skim milk). The following primary antibodies were purchased from Abcam and utilized as follows: CS (#ab96600, conc. 1:1000 5% BSA), β-HAD (#ab81492, conc. 1:1000 5% BSA). After incubation with primary antibodies, membranes were incubated with horseradish peroxidase-conjugated secondary antibody at RT for 1 hour in a 1:5000 TBST solution with 1% BSA (for COX IV a 1:10.000 solution was used). The proteins of interest were visualized with chemiluminescent substrate (#1705061, BIO-RAD, Hercules, CA, USA). Arbitrary protein intensity were quantified with an UVP imaging system (UVP, CA, USA) and normalized to the total amount of protein loaded in the corresponding lane using Stain Free Technology [25,26].

In the first series, data are presented as fold change and phosphorylated proteins are expressed relative to their non-phospohorylated from. In the second series, data are expressed as arbitrary units.

#### Statistical analysis

Data are presented as mean ± SEM, unless otherwise indicated. Statistical analyses of infarct size, myocardial glucose uptake during stabilisation and activation of signalling pathways were performed by one-way ANOVA. Two-way ANOVA with repeated measurements was used for hemodynamic function and myocardial glucose uptake during reperfusion. Bonferroni post hoc test was perfomed for multiple comparisons. Analyses of mitochondrial protein content were performed by t-tests. All statistical calculations were performed using GraphPad Prism (GraphPad Software, CA, USA). p≤0.05 was considered significant. The required sample size was estimated from previously published work using the isolated heart model [20].

## Results

### Animal characteristics

At age 3–4 months, the animals underwent treadmill tests, and the longest distance of three separate runs was lower in LCR than HCR rats (203±7 m vs 1905±51 m; p<0.01). At 8 months of age, LCR rats had a significantly higher body weight than HCR rats (463±6 g vs 384±7 g, p<0.01).

### Infarct size

**Fig 2:**
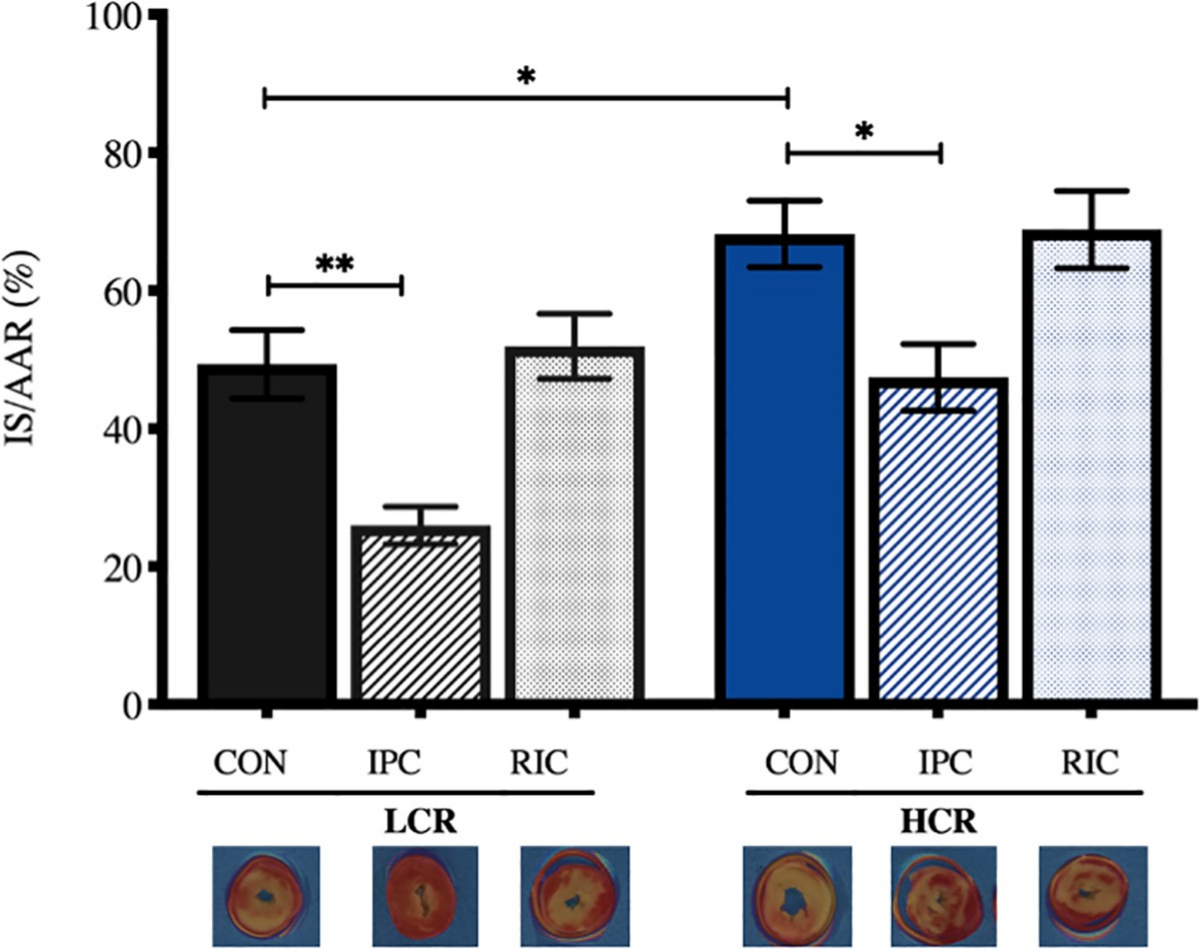
Infarct size. Ratio of infarct size over area at risk. IS: Infarct size, AAR: Area at risk, LCR: Low capacity runners, HCR: High capacity runners, CON: Control, IPC: Local ischemic preconditioning, RIC: Remote ischemic preconditioning. * p<0.05, ** p<0.01. n = 9–10. Values are presented as mean ± SEM.

LCR rats had lower IS than HCR rats (49±5% vs 68±5%, p = 0.04) (Fig 2). In both phenotypes, IPC reduced IS compared to controls (in LCR rats: 49±5% vs 26±3%, p<0.01; and HCR rats: 68±5% vs 47±5%, p = 0.01). RIC had no effect on IS in either LCR animals (49±5% and 52±5%, p>0.99) or HCR rats (68±5% and 69±6%, p>0.99).

### Hemodynamic function

#### Stabilisation

During stabilisation, LCR control rats had higher LVDP than HCR control rats (9 minutes: p<0.03, 29 minutes: p = 0.01) (Table 1). Rate pressure procuctn (RPP) was also higher in LCR control rats than in HCR controls (29 minutes: p<0.01). IPC reduced LVDP prior to index ischemia in LCR (p = 0.05) as well as HCR rats (p<0.01). RIC lowered heart rate (HR) in LCR (p = 0.03) but not in HCR rats. Coronary flow did not differ between groups.

**Table 1:** Hemodynamic function. Representative timepoints of hemodynamic function during stabilisation and reperfusion.

Stabilisation				Ischemia	Reperfusion		
		9 min	29 min		10 min	30 min	120 min
**Left ventricular developed pressure (mmHg)**							
	CON	189±4	177±3		20±3	57±10	50±5
**LCR**	IPC	190±5	126±19 †		42±10	90±8 †	69±3 ††
	RIC	180±5	172±4		22±4	46±7	39±5
	CON	160±9*	152±6 *		21±6	43±8	37±4
**HCR**	IPC	169±6	116±6 ##		25±7	64±7	50±7
	RIC	181±5	167±6		13±4	43±8	35±4
**Heart rate (beats/min)**							
	CON	231±11	270±19		223±14	265±16	288±15
**LCR**	IPC	213±16	213±17		278±26	285±22	355±53
	RIC	235±8	210±12 Ψ		229±18	269±18	281±18
	CON	235±22	228±16		195±18	213±20	240±12
**HCR**	IPC	239±12	225±12		194±13	209±11	251±13
	RIC	217±12	224±12		233±14	219±7	243±12
**Rate pressure product (beats/min×mmHg)**							
	CON	43613±1891	47271±2465		4351±850	14426±2269	14458±1629
**LCR**	IPC	40756±3389	26842±4208††		10972±2896	24792±2300 †	24940±4254
	RIC	42560±2044	36320±2334 Ψ		4622±868	12449±2050	11445±1703
	CON	37636 ±4269	34338±2277 **		4345±1609	9357±1916	9091±1391
**HCR**	IPC	40396±2503	25931±1641 #		4821±1355	13386±1557	12494±1819
	RIC	39161±2505	37184±2154		2902±1026	9356±1765	8308±1087
**Coronary flow (mL/min)**							
	CON	19±3	18±2		16±2	14±2	9±2
**LCR**	IPC	17±1	18±1		17±2	15±2	11±2
	RIC	15±1	14±1		13±1	12±1	8±1
	CON	19±3	18±2		16±2	14±2	9±2
**LCR**	IPC	17±1	18±1		17±2	15±2	11±2
	RIC	15±1	14±1		13±1	12±1	8±1

LCR: Low capacity runners, HCR: High capacity runners, CON: Control, IPC: Local ischemic preconditioning, RIC: Remote ischemic preconditioning, min: Minutes.

During stabilisation two single timepoints were used: before IPC (9 min) and after induction of IPC (29 min). During reperfusion three timepoints were used: 10, 30 and 120 minutes of reperfusion. Statistical analyses of reperfusion were performed as ANOVA with repeated measurements.

* represents comparison between LCR CON and HCR CON

† Represents comparison between LCR CON and LCR IPC

Ψ represents comparison between LCR CON and LCR RIC

# represents comparison between HCR CON and HCR IPC. One symbol p ≤ 0.05, two symbols: p ≤ 0.01.

n = 9–10. Values are presented as mean ± SEM.

#### Reperfusion

During reperfusion, LVDP, HR and RPP were similar in LCR and HCR control rats (Table 1). IPC increased LVDP at both 30 minutes (p = 0.04) and 120 minutes (p<0.01) in LCR rats, whereas the increment in HCR rats was not statistically significant. IPC also improved RPP at 30 minutes in LCR rats (p = 0.02). RIC did not change LVDP or RPP in either LCR or HCR rats.

### Enzymes involved in substrate metabolism and mitochondrial proteins

**Fig 3:**
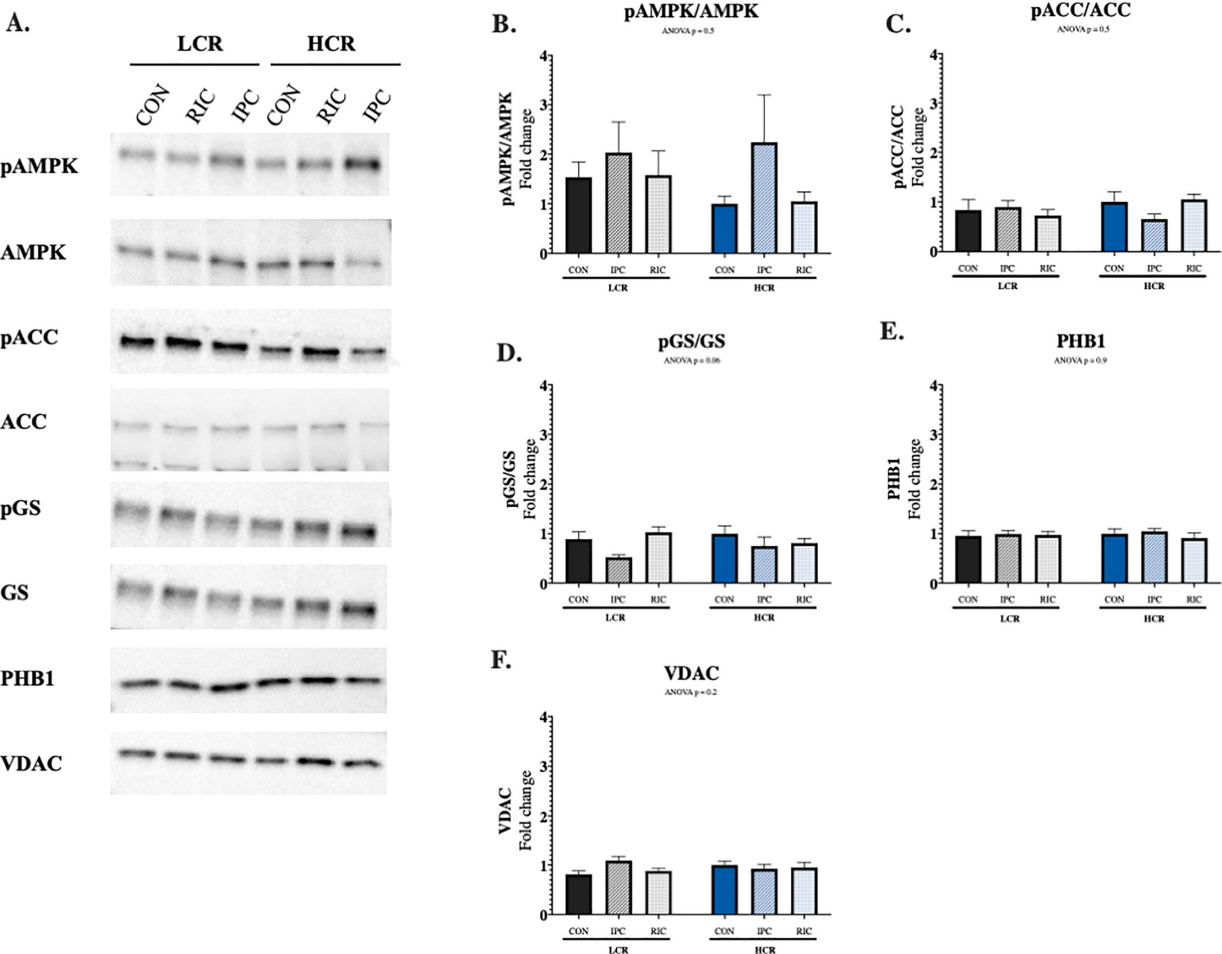
Activation of signalling pathways. (A) Representative blots, (B) Phosphorylation of AMPK, (C) Phosphorylation of ACC, (D) Phosphorylation of GS, (E) levels of PHB1 and (F) Levels of VDAC. LCR: Low capacity runners, HCR: High capacity runners, CON: Control, IPC: Local ischemic preconditioning, RIC: Remote ischemic preconditioning, AMPK: AMP-activated Kinase, ACC: Acetyl-CoA Carboxylase, GS: Glycogen Synthase, PHB1: Prohibitin-1, VDAC: Voltage-dependent anion channel, COX IX: Cytochrome c oxidase IX, β-HAD: β-hydroxyacyl-CoA dehydrogenase and CS: Citrate synthase. Values are presented as mean ± SEM. N = 5–8.

The ratio of pAMPK/AMPK did not differ significantly between LCR and HCR control rats (Fig 3). IPC did not change the pAMPK/AMPK ratio in LCR rats. IPC increased the pAMPK/AMPK ratio two-fold in HCR rats, but the increment did not reach statistically significance (ANOVA p = 0.6). RIC did not alter the pAMPK/AMPK ratio.

The absence of AMPK activation was supported by similar ACC and GS phosphorylation in LCR and HCR animals, and none of the regulators were affected by IPC or RIC. Similarly, the mitochondrial proteins PHB1 and VDAC were not affected by animal type or IPC and RIC.

### Myocardial glucose uptake

#### Stabilisation

**Fig 4:**
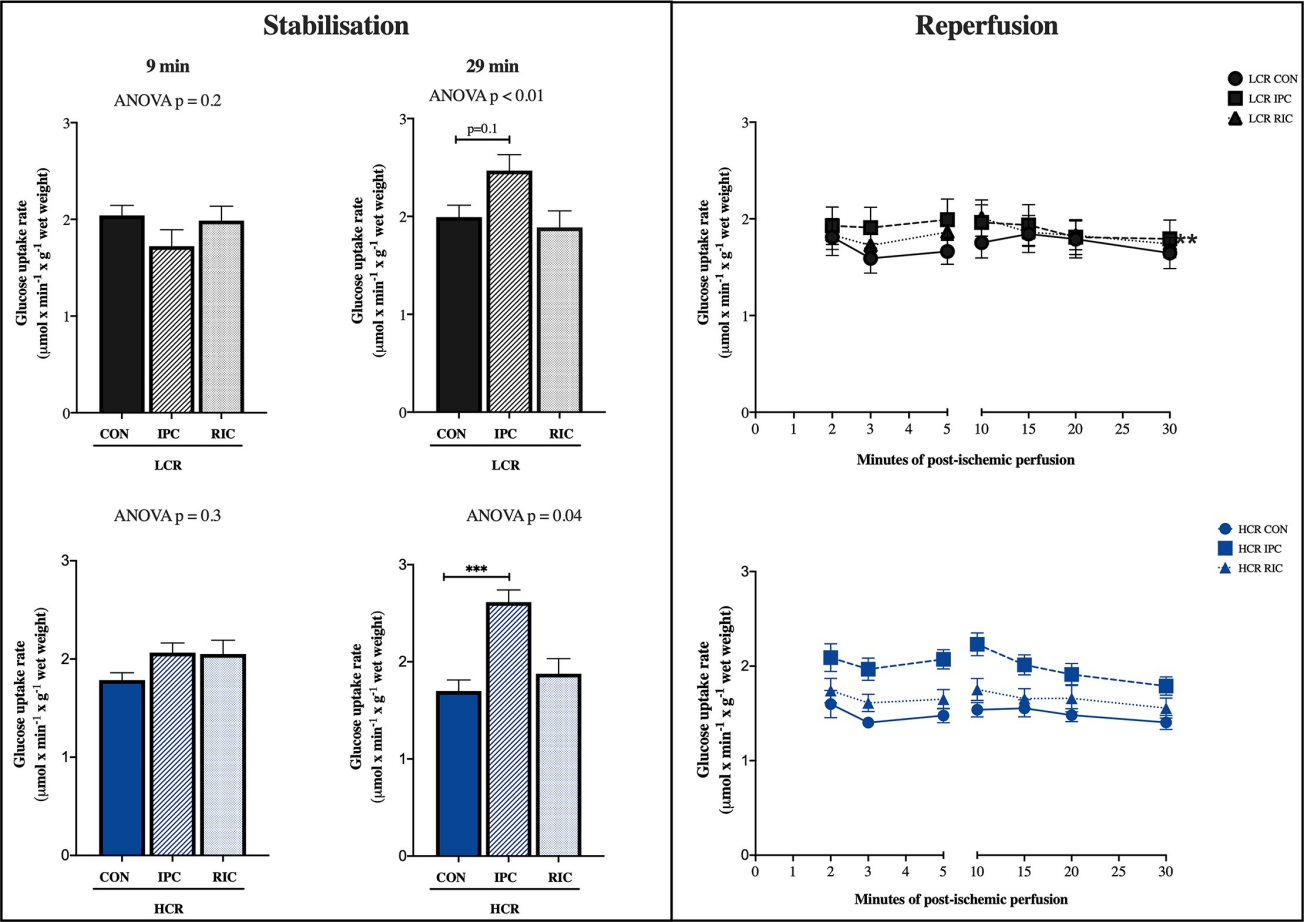
Myocardial glucose uptake. During stabilisation two sample points are displayed, one before IPC (9 min) and one after induction of IPC (29 min). During reperfusion samples are displayed over time. LCR: Low capacity runners, HCR: High capacity runners, CON: Control, IPC: Local ischemic preconditioning, RIC: Remote ischemic preconditioning, min: Minutes. * p<0.05, ** p<0.01, *** p<0.001. n = 5–9. Values are presented as mean ± SEM.

Myocardial glucose uptake was similar in all groups during stabilisation (Fig 4).

IPC increased myocardial glucose uptake in LCR (p = 0.10) and HCR rats, but only statistically significantly in HCR rats (p<0.01). RIC did not change the glucose uptake.

#### Reperfusion

During reperfusion myocardial glucose uptake was higher in LCR controls than in HCR control rats (p<0.01). IPC did not change the uptake in LCR rats. IPC increased myocardial glucose uptake in HCR animals (p<0.01) (Fig 4).

### Basal expression of selected mitochondrial proteins

**Fig 5:**
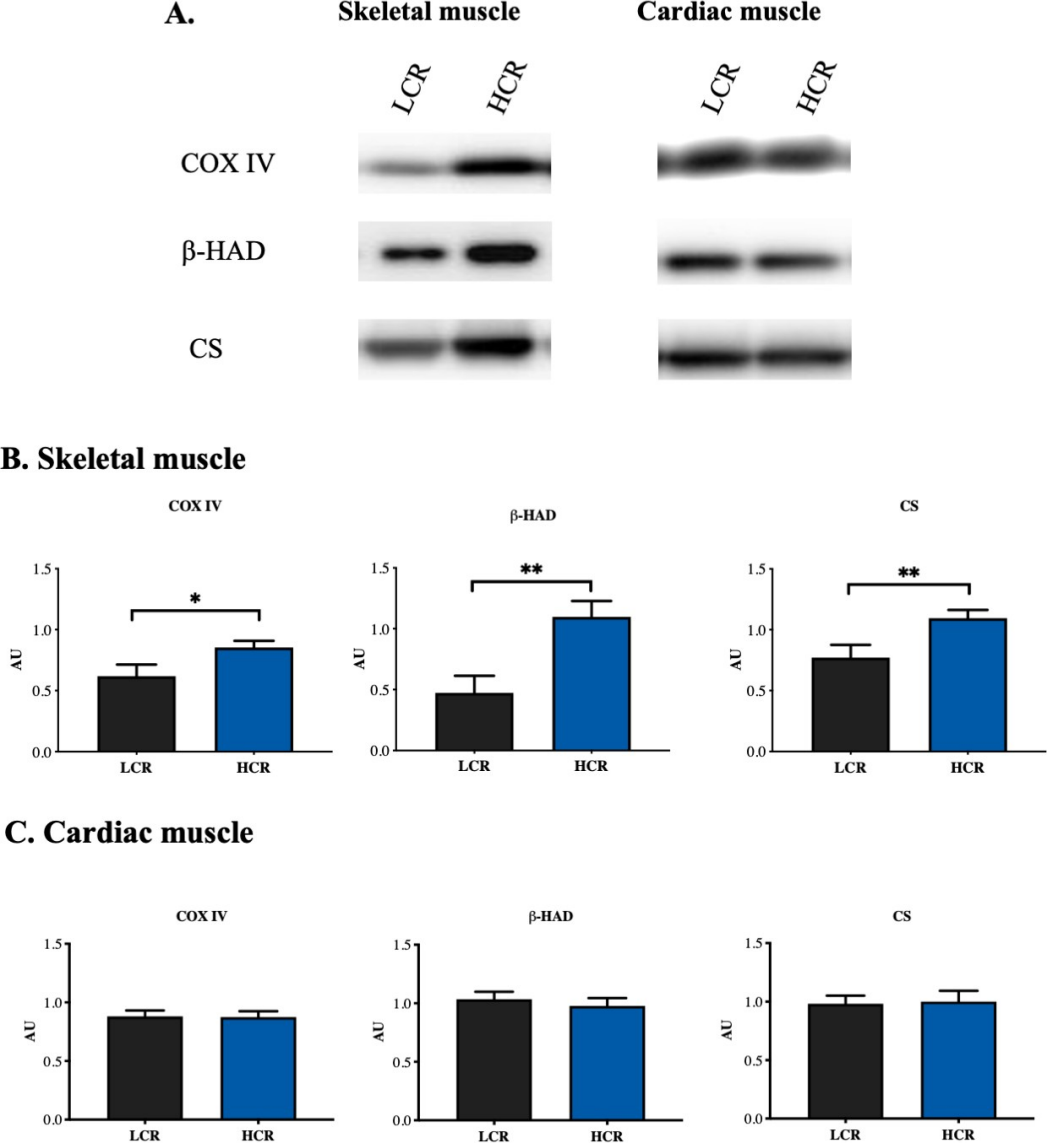
Expression of selected mitochondrial proteins. (A) Representative blots for all groups. (B) Mitochondrial protein analyses on skeletal muscle and (C) cardiac muscle. HCR: High capacity runners, LCR: Low capacity runners, COX IV: Cytochrome c oxidase, complex IV, β-HAD: β-hydroxyacyl-coenzyme dehydrogenase, CS: Citrate synthase. * p<0.05, ** p<0.01. n = 8 in all groups. Values are presented as mean ± SEM.

COX IV, β-HAD and CS were measured in control rats. The mitochondrial markers COX IV, β-HAD and CS in tibialis anterior muscle were lower in LCR rats than in HCR rats (p<0.05, p<0.01 and p<0.01, respectively) (Fig 5B). In cardiac muscle there were no differences in content of any of the mitochondrial proteins (Fig 5C).

## Discussion

The main finding of the present study is that LCR rats are less sensitive to IR injury than HCR rats, indicating that aerobic capacity may influence endogenous sensitivity to IR injury. Despite this difference, the efficacy of IPC was preserved and equal in both phenotypes, indicating that the cardioprotective potential was independent of differences in aerobic capacity. RIC was not effective in either phenotype and may reflect a challenge in translating the effect of RIC to an older rat model [27].

Here, we used a rat model that was derived from a genetically heterogeneous stock (N/NIH) and developed by selective breeding based on running capacity as a surrogate marker of aerobic capacity (16). Through generations of selective breeding, two distinct phenotypes have evolved [18]. LCR rats have progressed to a phenotype of metabolic syndrome including obesity, elevated blood glucose, and high levels of insulin, triglycerides and free fatty acids [15]. In contrast, HCR rats have developed to become resistant to development of risk factors such as diet-induced obesity and insulin resistance [28]. The favourable phenotype in HCR animals has been linked to longevity, as life expectancy is increased by 45% in HCR compared to LCR [18]. It may be unexpected that infarct sizes in HCR rats were larger than in LCR rats because cardiac IR injury is usually attenuated in models with exercise-trained animals with high aerobic capacity [29–32]. The high intrinsic aerobic capacity of HCR rats is a result of a primarily genetic phenotype rather than characteristic of the exercise trained animal models [33]. During running, cardiac gene expression of HCR rats shows a preference for lipid metabolism in contrast to a preferential carbohydrate metabolism in LCR hearts [34]. Carbohydrates are well known preferential substrates in the heart during IR because of its superior energy efficiency [35,36]. The altered metabolic phenotype, including a state of metabolic syndrome, is a confounding factor in LCR rats. The effect of the metabolic disarray may be close to a state of early onset type-2 diabetes mellitus with activation of endogenous cardioprotective mechanisms [20,37].

Our results are consistent with the findings by Høydal et al, who demonstrated similar or even worse cardiac- and cardiomyocyte contractile function following myocardial infarction in HCR rats compared to LCR rats, measured by echocardiography and isolated perfused heart evaluation [38]. Hussain et al evaluated the cardiac output in LCR and HCR rats at baseline and after IR injury in an isolated working heart model. In contrast to our results, they demonstrated no difference in restoration of cardiac output following IR in rats at the same age as ours [39]. The rats in the study by Hussain et al. originated from an early breeding generation (3rd), and the hemodynamic performance during stabilisation of the LCR vs HCR rats did not correspond with our results. During stabilisation, we observed that the LCR rats produced a higher RPP than HCR rats in the absence of any HR differences, whereas Hussain et al found higher cardiac output in HCR than in LCR rats [39]. Others, who have investigated isolated hearts from HCR and LCR rats of a younger age found no differences in cardiac function [40]. Hence, both animal age and advancement of phenotype may influence cardiac function.

The cardioprotective effect of IPC is consistent and reproducible in all animal species studied until now. The efficacy is in the range of 25–45% IS reduction depending on the experimental setting, animal strain, and duration of ischemia [41,42]. We extend this finding by demonstrating that the efficacy of IPC is similar in LCR and HCR rats irrespective of the intrinsic difference in sensitivity to IR injury. In contrast, RIC did not yield cardioprotection in either LCR or HCR rats. RIC seems to induce a weaker stimulus than IPC, but still yields cardioprotection across strains in *in vivo* and *in vitro* models [43]. Most previous studies use healthy rats of 2–4 months of age, we used rats at the age of 8 months, which could influence the response [42]. A limitation of our study is that we did not study the influence of a more powerful RIC stimulus. Finally, diabetes is known to attenuate the response to mechanical conditioning strategies [44–46], and this may also apply to LCR animals.

LCR hearts produced enhanced cardiac work, with increased LVDP during stabilisation, while there was no difference in glucose uptake compared to HCR. This finding could indicate a more efficient handling of glucose in the LCR animals, possibly by the upregulated carbohydrate metabolism in LCR rats as described by Bye et al [34]. IPC increases glucose uptake during stabilisation [36]. We documented this finding in HCR animals whereas the increase in glucose uptake during stabilisation was not statistically significant in LCR hearts. During reperfusion IPC only increased glucose uptake in HCR hearts. Considering the similar degree of infarct size reduction, the difference in glucose handling between HCR and LCR did not seem to influence the effect of IPC induced cardioprotection.

IPC as well as exercise induce cardioprotection by AMPK activation [47–49], such that IPC and exercise may interact and modify the cardioprotective capability. We investigated preischemic activation of both AMPK and the downstream AMPK target ACC. In addition, we evaluated GS phosphorylation at S641, an inhibiting site phosphorylated by GSK3. However, we observed no difference in the cardioprotective capacity by IPC between LCR and HCR rats. Correspondingly, baseline AMPK activation and upregulation by IPC as well as downstream targets, including ACC and GS, were similar in LCR and HCR rats. Our findings are in accordance with previous results [50], supporting that the differences in IS between HCR and LCR hearts may not depend on this signalling pathway.

The cardiovascular health benefits of physical activity are multifactual mediated by a favorable endocrine milieu [51]. IL-6 is a pro-inflammatory cytokine. Release of IL-6 by muscles during exercise mediates anti-inflammatory effects by increasing the levels of an IL-1 receptor antagonist and IL-10 [52]. Up to our experiments, the LCR and HCR animals in our study were not subjected to any kind of structured exercise that might increase the circulating levels of myokines prior to the ischemia reperfusion exposure. However, the spontaneous activity level is higher in HCR than LCR animals [53]. Levels of circulating IL-10 are higher in LCR than HCR animals, but not preceeded by an increase in IL-6 [18]. IL-10 has cardioprotective potential [54]. Differences in circulating IL-10 in LCR and HCR animals were minor and whether the anti-inflammatory effect of IL-10 interacts with the sensitivity to IR injury needs further investigation.

Aerobic capacity is tightly linked to mitochondrial numbers and function, and in the heart IPC modifies the deleterious effects of IR injury on mitochondria. In skeletal muscle, increased content of mitochondrial oxidative enzymes are a classic marker of adaptation to increased physical activity [55]. Our observation of increased content of three mitochondrial proteins in skeletal muscle therefore verified the increased physical activity in HCR rats. In contrast, the content of the same mitochondrial proteins in the heart was not increased in HCR rats compared to LCR rats, which is consistent with observations by Souza et al [56]. Interestingly, Souza et al observed only modest differences in mitochondrial respiratory rates which could suggest that the differences in sensitivity to IR injury between HCR and LCR rats is not due to variances in baseline myocardial mitochondrial function. Whether the mitochondria from HCR and LCR rats react differently during IR is not clear from our data.

Patients suffering from metabolic syndrome or diabetes mellitus have aggravated outcome after acute myocardial infarction [57]. While this counteracts the findings of this study, our results may be linked to the “obesity paradox”, showing that in heart failure cohorts obese patients have a better clinical condition compared to lean patients [58].

### Limitations

The use of an *in vitro* rat model limits transferability to human physiology. *In vitro* induction of IPC may potentially lack *in vivo* elements of IPC including the neurohumoral response. We aimed to study the impact of exercise in the most powerful conditioning modality, which is the *in vitro* setting of IPC. When evaluating myocardial energy metabolism and signal transduction we are limited by glucose as the only available substate and rapid metabolic changes, such that we may have missed windows of opportunity to detect rapid temporary changes.

## Conclusion

Sensitivity to IR injury is associated with differences in intrinsic aerobic capacity but the cardioprotective efficacy of IPC is unchanged, while RIC is not effective in either HCR or LCR. The cardioprotection in LCR rats is not associated with measurable differences in myocardial glucose uptake, AMPK activation and mitochondrial protein content prior to onset of ischemia.

## Supporting information

S1 Fig Infarct size in Sprague Dawley rats. Ratio of infarct size over area at risk. IS: infarct size, AAR: area at risk, CON: control, IPC: local ischemic preconditioning, RIC: remote ischemic preconditioning. * p<0.05, ** p<0.01. n = 4–5. Values are presented as mean ± SEM.
(DOCX)

S2 Fig Glucose uptake in Sprague Dawley rats. During stabilisation two sample points are displayed, one before IPC (9 min) and one after induction of IPC (29 min). During reperfusion samples are displayed over time. CON: control, IPC: local ischemic preconditioning, RIC: remote ischemic preconditioning, min: minutes. * p<0.05, ** p<0.01, *** p<0.001. n = 4–5. Values are presented as mean ± SEM.
(DOCX)

S1 Raw images (PDF)
