# Supplementary material for: Differences in intrinsic aerobic capacity alters sensitivity to ischemia-reperfusion injury but not cardioprotective capacity by ischemic preconditioning in rats
Source: PLoS One. 2020 Oct 27;15(10):e0240866. doi: 10.1371/journal.pone.0240866 (PMC7591019; doi:10.1371/journal.pone.0240866)
Supplement: S1 Fig — Ratio of infarct size over area at risk. IS: infarct size, AAR: area at risk, CON: control, IPC: local ischemic preconditioning, RIC: remote ischemic preconditioning. * p<0.05, ** p<0.01. n = 4–5. Values are presented as mean ± SEM. (DOCX) [file pone.0240866.s001.docx]

**Description of pilot trials**

Before performing the experiments with the HCR and LCR rats, we performed some of the analyses in 6 months old Sprague Dawley rats. The pilot trials were performed to test the cardioprotective modalities in animals older than we usually use, and validate the glucose uptake analyses.

The results of infarct size and glucose uptake analyses from these initial pilot trials in 6 months old Sprague Dawley rats are presented below:

**Supplementary results**

Infarct size:

In Sprague Dawley rats, IPC reduced IS compared to controls (50±6% vs 76±6%, p<0.01) (Fig S1). RIC did not significantly reduce IS (67±3% vs 76±6%, p=0.47).

**Figure S1. Infarct size in Sprague Dawley rats.** Ratio of infarct size over area at risk. IS: infarct size, AAR: area at risk, CON: control, IPC: local ischemic preconditioning, RIC: remote ischemic preconditioning. * p<0.05, ** p<0.01. n=4-5. Values are presented as mean ± SEM.
