# Supplementary material for: Differences in intrinsic aerobic capacity alters sensitivity to ischemia-reperfusion injury but not cardioprotective capacity by ischemic preconditioning in rats
Source: PLoS One. 2020 Oct 27;15(10):e0240866. doi: 10.1371/journal.pone.0240866 (PMC7591019; doi:10.1371/journal.pone.0240866)
Supplement: S2 Fig — During stabilisation two sample points are displayed, one before IPC (9 min) and one after induction of IPC (29 min). During reperfusion samples are displayed over time. CON: control, IPC: local ischemic preconditioning, RIC: remote ischemic preconditioning, min: minutes. * p<0.05, ** p<0.01, *** p<0.001. n = 4–5. Values are presented as mean ± SEM. (DOCX) [file pone.0240866.s002.docx]

Glucose uptake:

In Sprague Dawley rats, IPC increased glucose uptake compared to controls (p<0.001) (Fig S2). RIC did not increase glucose uptake. During reperfusion the IPC group maintained the increase in glucose uptake (p<0.001).

**Figure S2. Glucose uptake in Sprague Dawley rats.** During stabilisation two sample points are displayed, one before IPC (9 min) and one after induction of IPC (29 min). During reperfusion samples are displayed over time.

CON: control, IPC: local ischemic preconditioning, RIC: remote ischemic preconditioning, min: minutes.

* p<0.05, ** p<0.01, *** p<0.001. n=4-5. Values are presented as mean ± SEM.

Infarct size was significantly reduced by IPC in the 6 months old animals, which corresponds to the main results from the HCR and LCR animals. We saw a numerical reduction in infarct size by RIC in the pilot trials, which did not reach significance, most likely due to low number of animals. In the HCR and LCR rats we did not see a similar reduction.

Glucose uptake was increased in IPC animals in the pilot trials, and the increased level was maintained during reperfusion. The increase in glucose uptake by IPC was more pronounced in the Sprague Dawley rats than in HCR and LCR rats, validation the method compared to earlier studies.
