# Supplementary material for: Differences in intrinsic aerobic capacity alters sensitivity to ischemia-reperfusion injury but not cardioprotective capacity by ischemic preconditioning in rats
Source: PLoS One. 2020 Oct 27;15(10):e0240866. doi: 10.1371/journal.pone.0240866 (PMC7591019; doi:10.1371/journal.pone.0240866)
Supplement: S1 Raw images — (PDF) [file pone.0240866.s003.pdf]

SUPPLEMENTAL MATERIAL – ORIGINAL RAW IMAGES

Differences in intrinsic aerobic capacity alters sensitivity to ischemia-reperfusion injury but not cardioprotective capacity by ischemic preconditioning in rats

Marie Vognstoft Hjortbak, Thomas Skjærlund Grønnebæk, Nichlas Riise Jespersen, Thomas Ravn Lassen, Jacob Marthinsen Seefeldt, Pernille Tilma Tonnesen, Rebekka Vibjerg Jensen, Lauren Gerard Koch, Steven L. Britton, Michael Pedersen, Niels Jessen, Hans Erik Bøtker

Original western blots

In the following sections we have provided the raw, original blots used for figures 3 and 5. The membranes were cut into strips for incubation of primary antibodies (after visualization of total protein content) based on the manufactures information of molecular weight of the individual proteins and our own and other laboratories previous experiments confirming specificity. This is standard procedure allowing for detection of multiple proteins from the same gel. Signals were detected using chemiluminescent substrate and quantified with digital imaging software. We have enclosed uncropped, unadjusted original blots of the membrane strips as requested

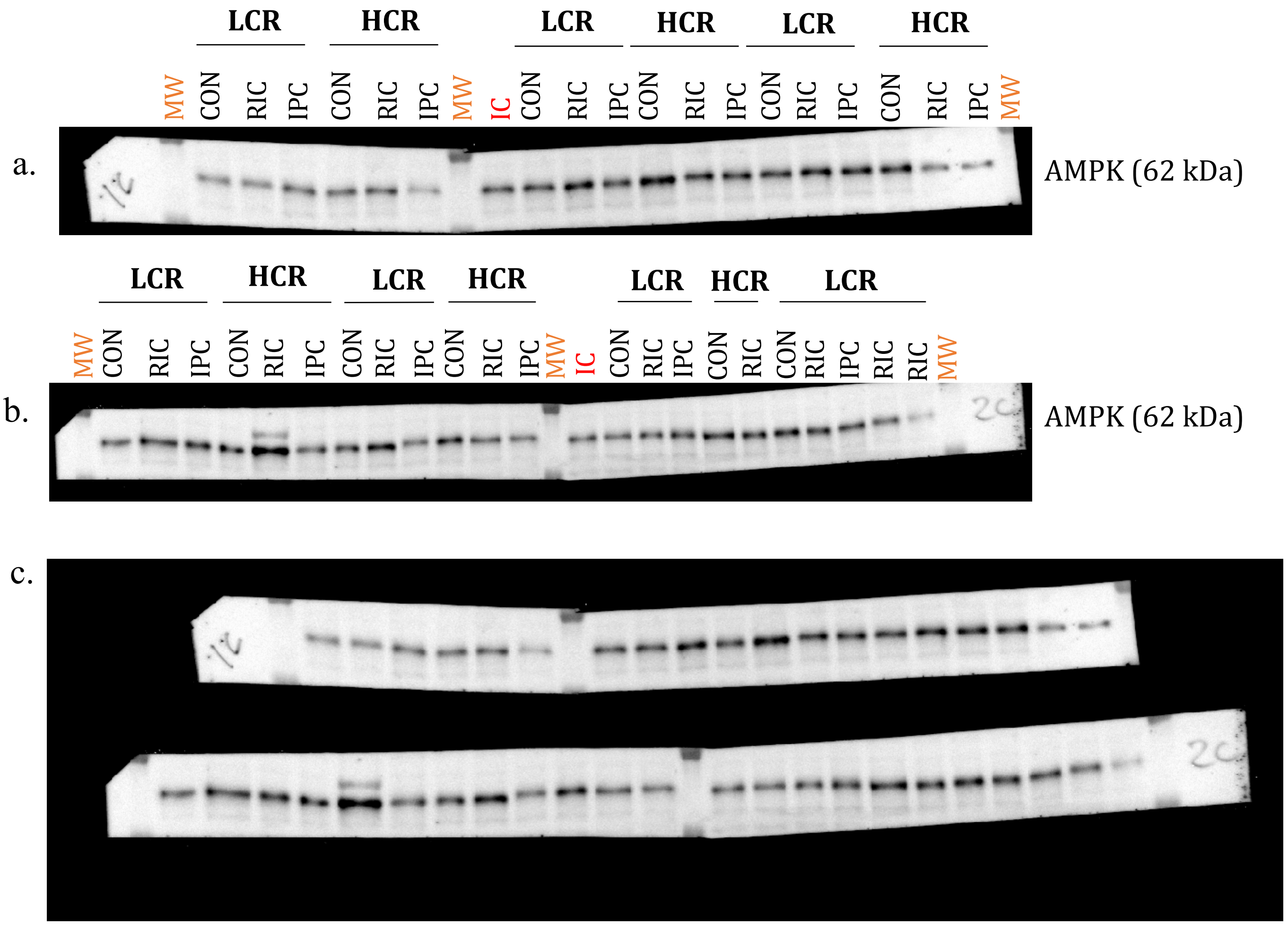

S3. Original blots for total AMPK. a. and b shows the bands separately, with makings, c. shows the total uncropped image. Results are shown in figure 3B.
MW: molecular weight marker. IC: internal control.

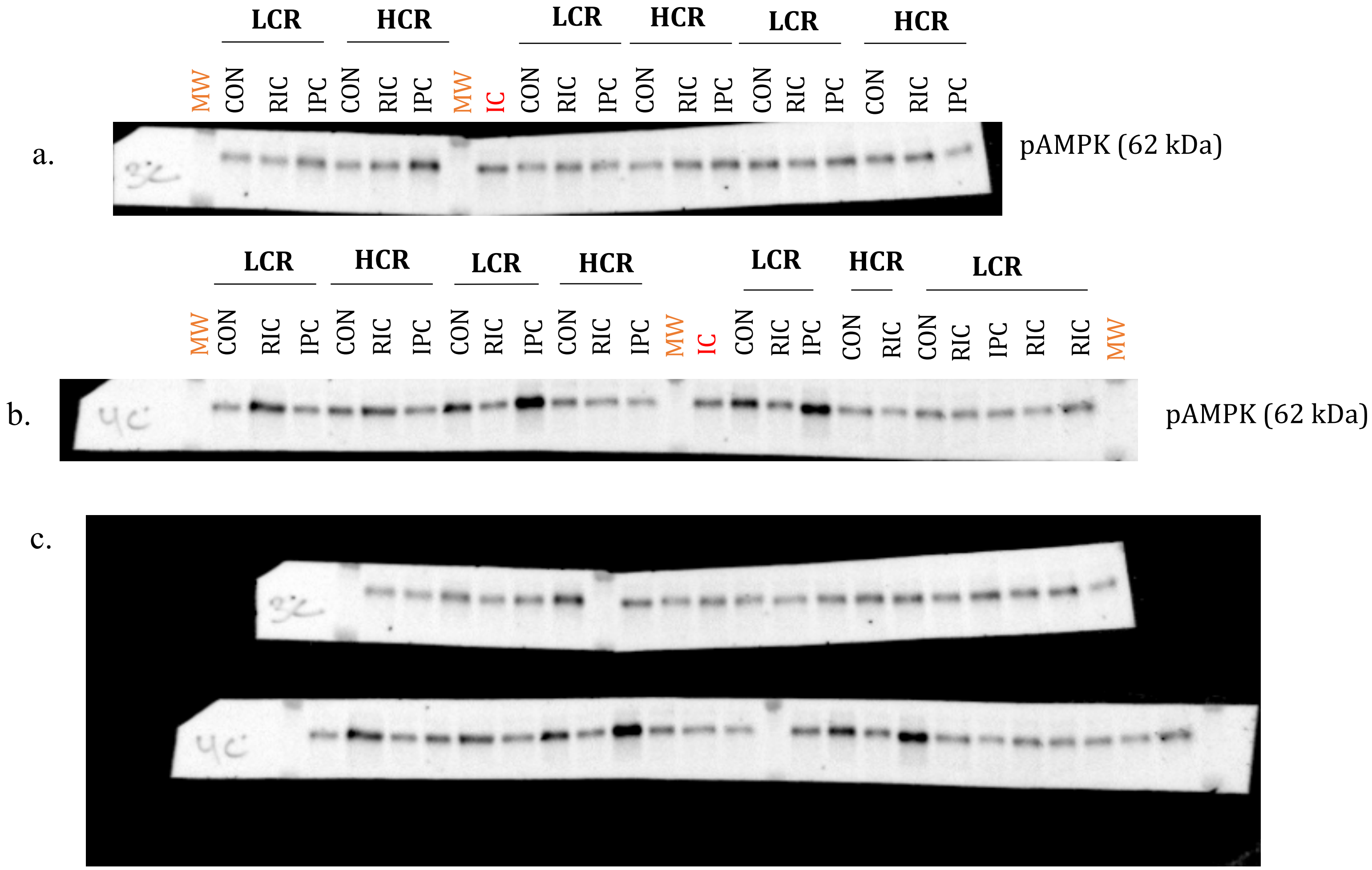

S4. Original blots for total pAMPK. a. and b shows the band separately, with makings, c. shows the total uncropped image. Results are shown in figure 3B.
MW: molecular weight marker. IC: internal control.

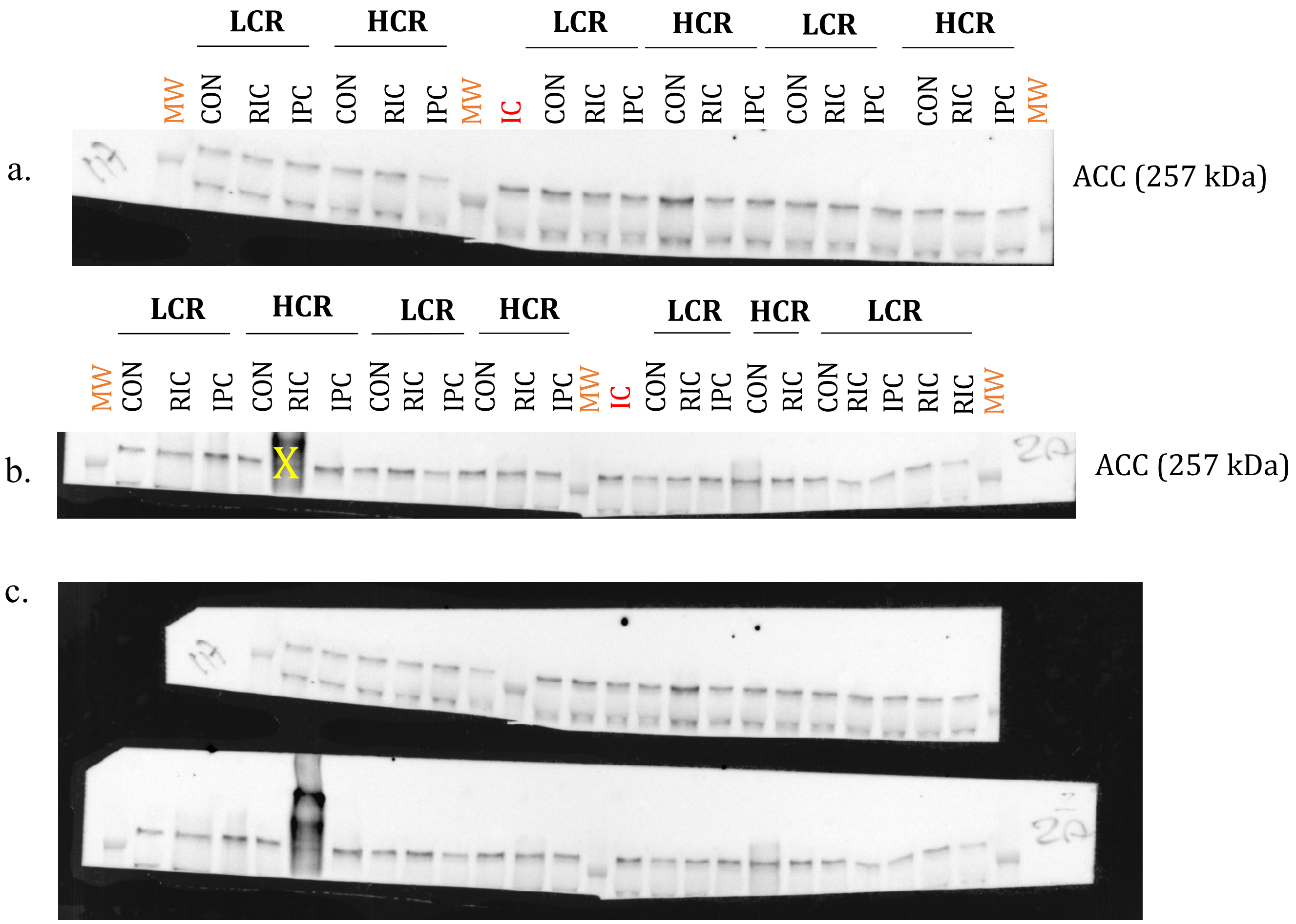

S5. Original blots for total ACC.. a. and b shows the band separately, with makings, c. shows the total uncropped image. Results are shown in figure 3C.
MW: molecular weight marker. IC: internal control.
X sample excluded from analysis.

a.

b.

c.

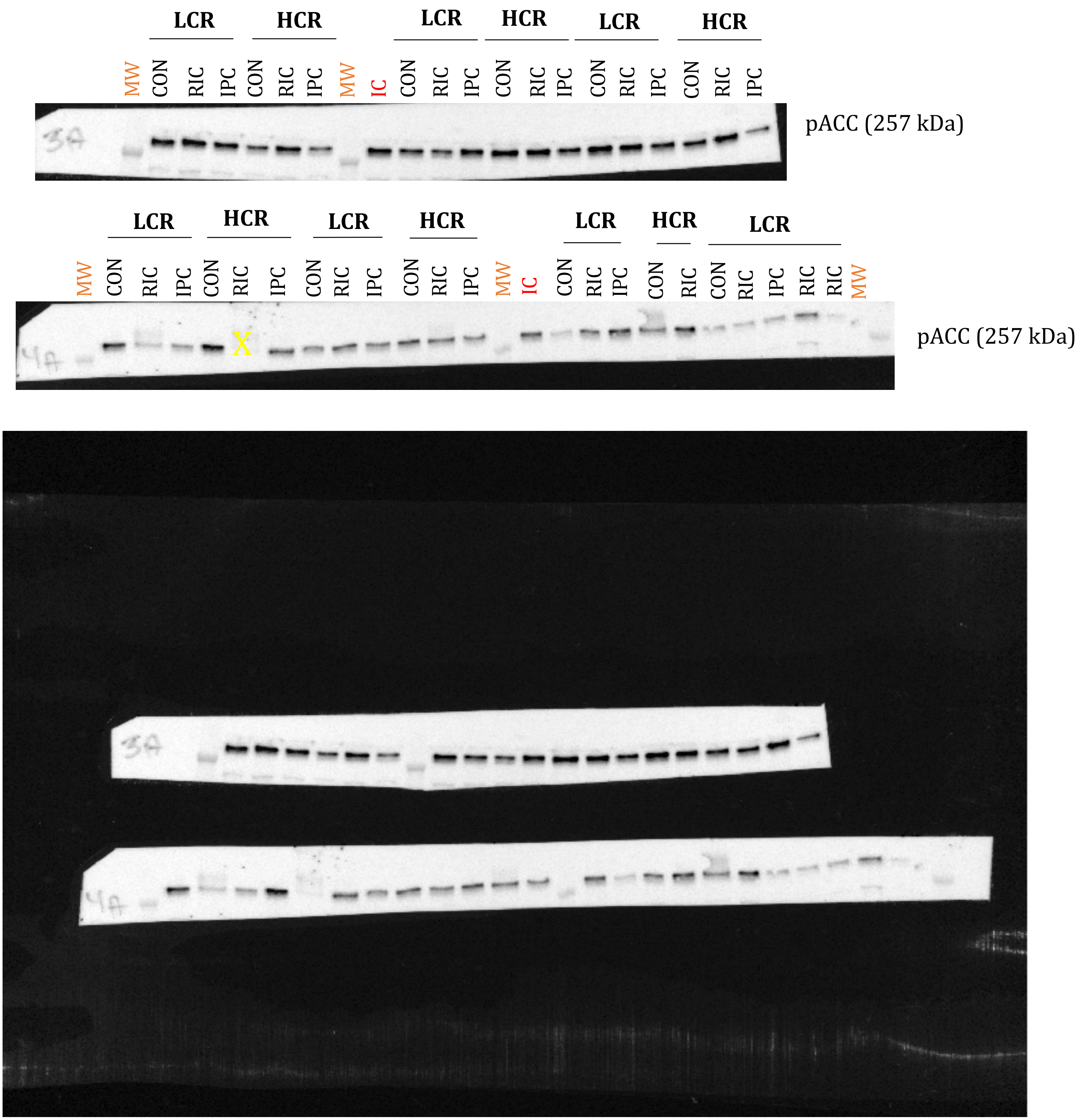

S6. Original blots for total pACC. a. and b shows the bands separately, with makings, c. shows the total uncropped image. Results are shown in figure 3B.
MW: molecular weight marker. IC: internal control.
X sample excluded from analysis.

a.

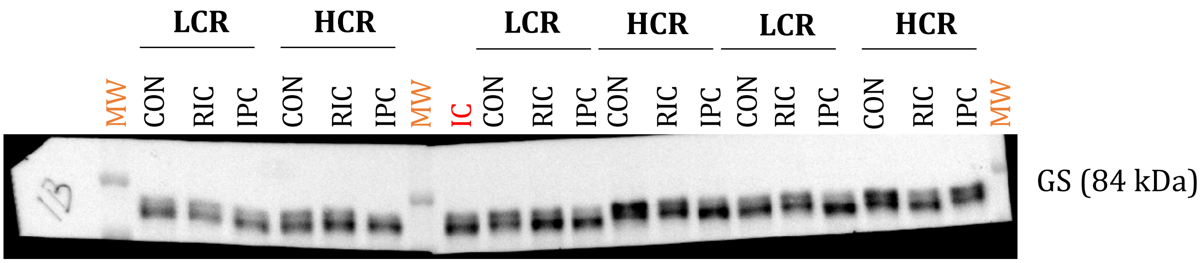

b.

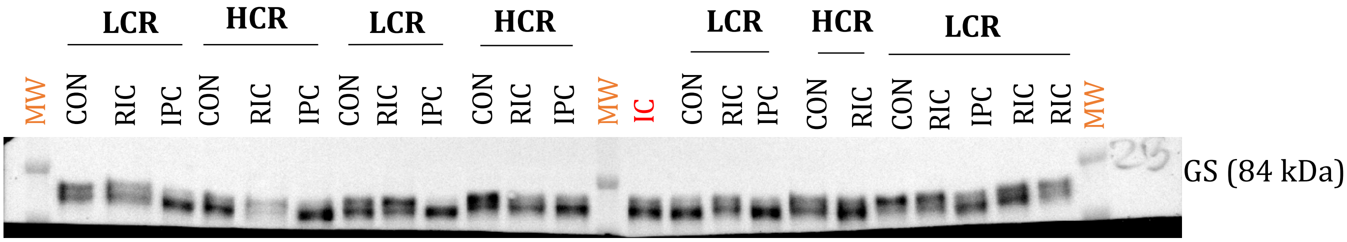

c.

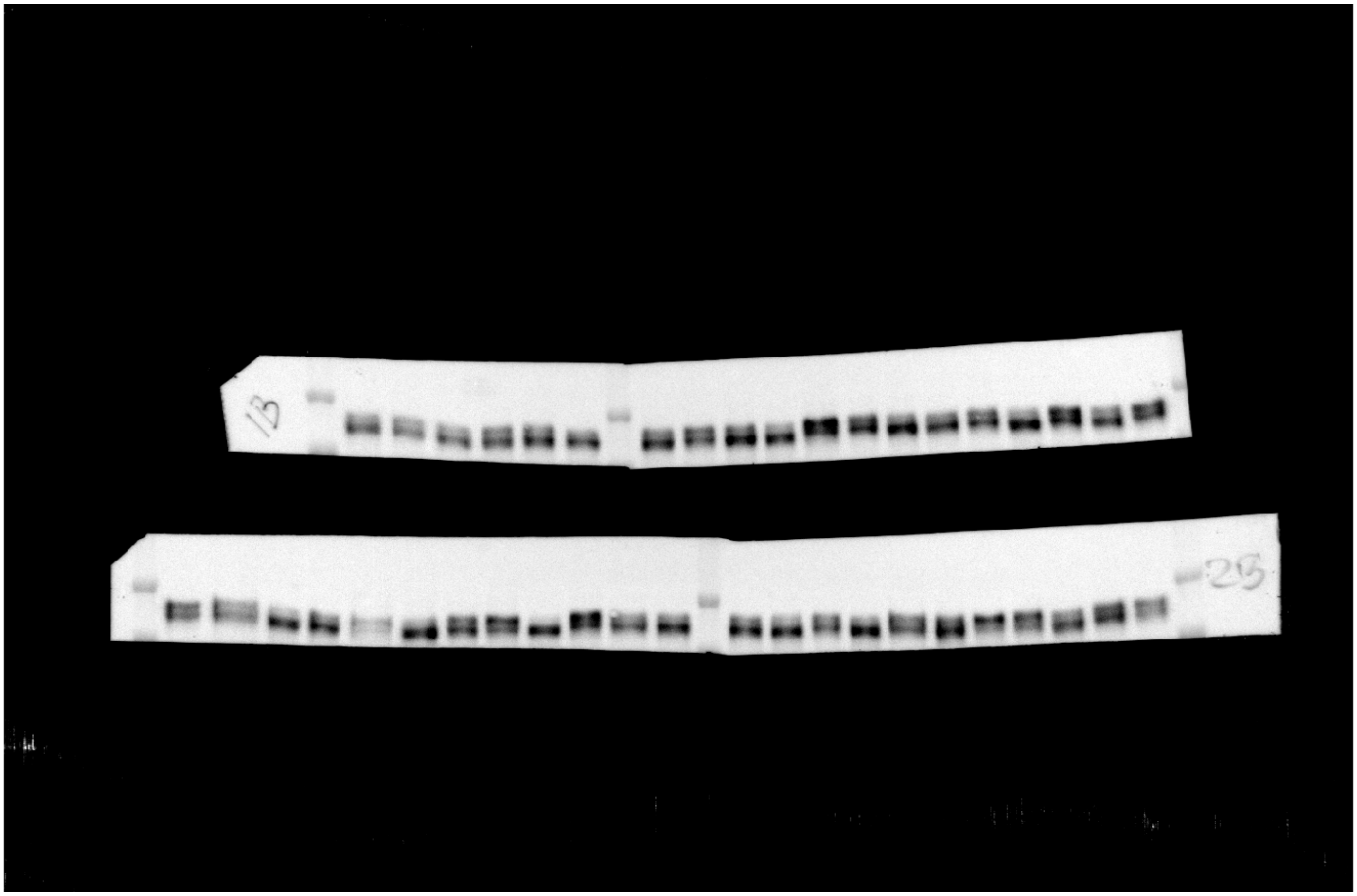

S7. Original blots for total GS. a. and b shows the bands separately, with makings, c. shows the total uncropped image. Results are shown in figure 3D.
MW: molecular weight marker. IC: internal control.

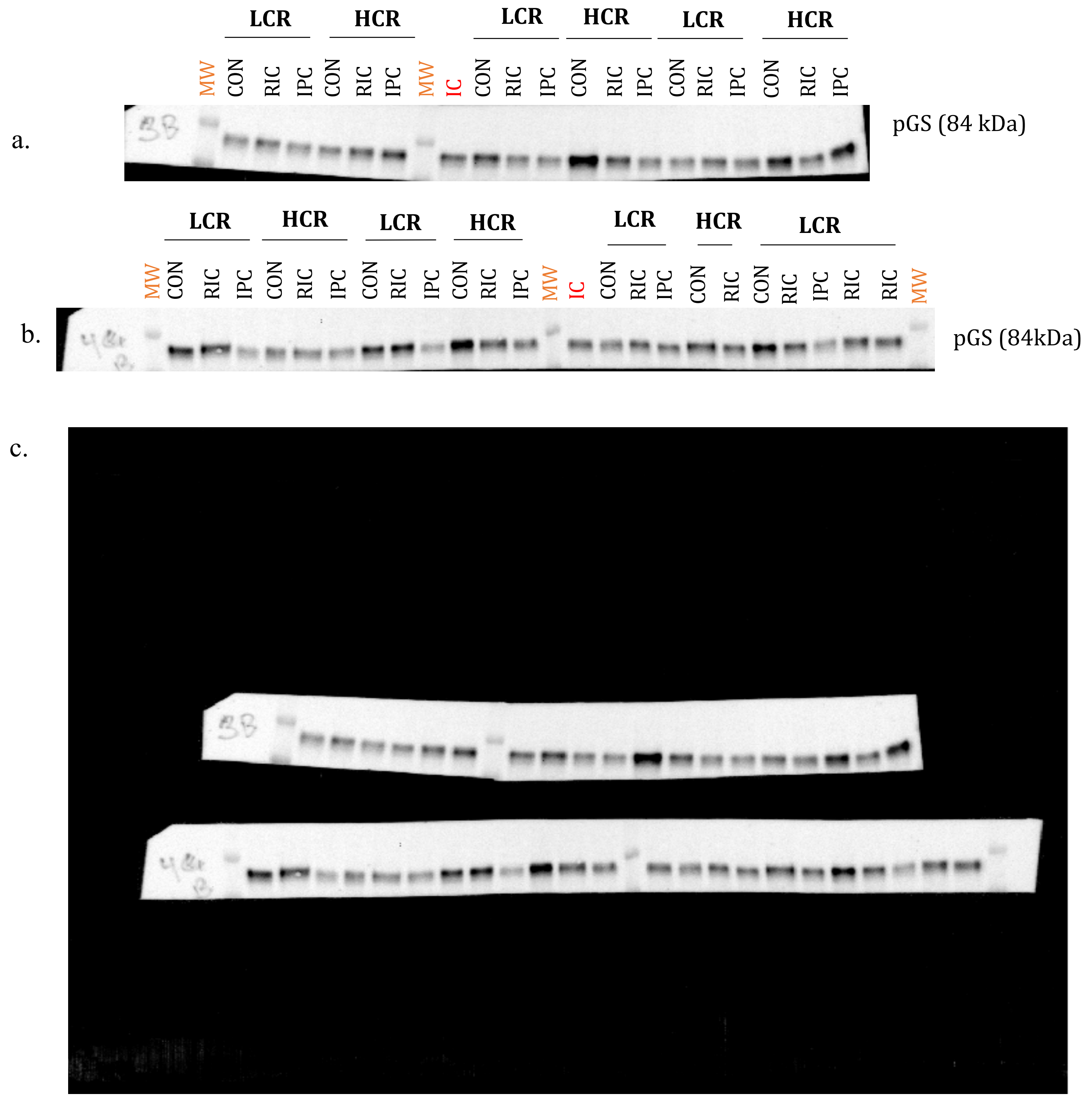

S8. Original blots for pGS. a. and b shows the bands separately, with makings, c. shows the total uncropped image. Results are shown in figure 3D.
MW: molecular weight marker. IC: internal control.

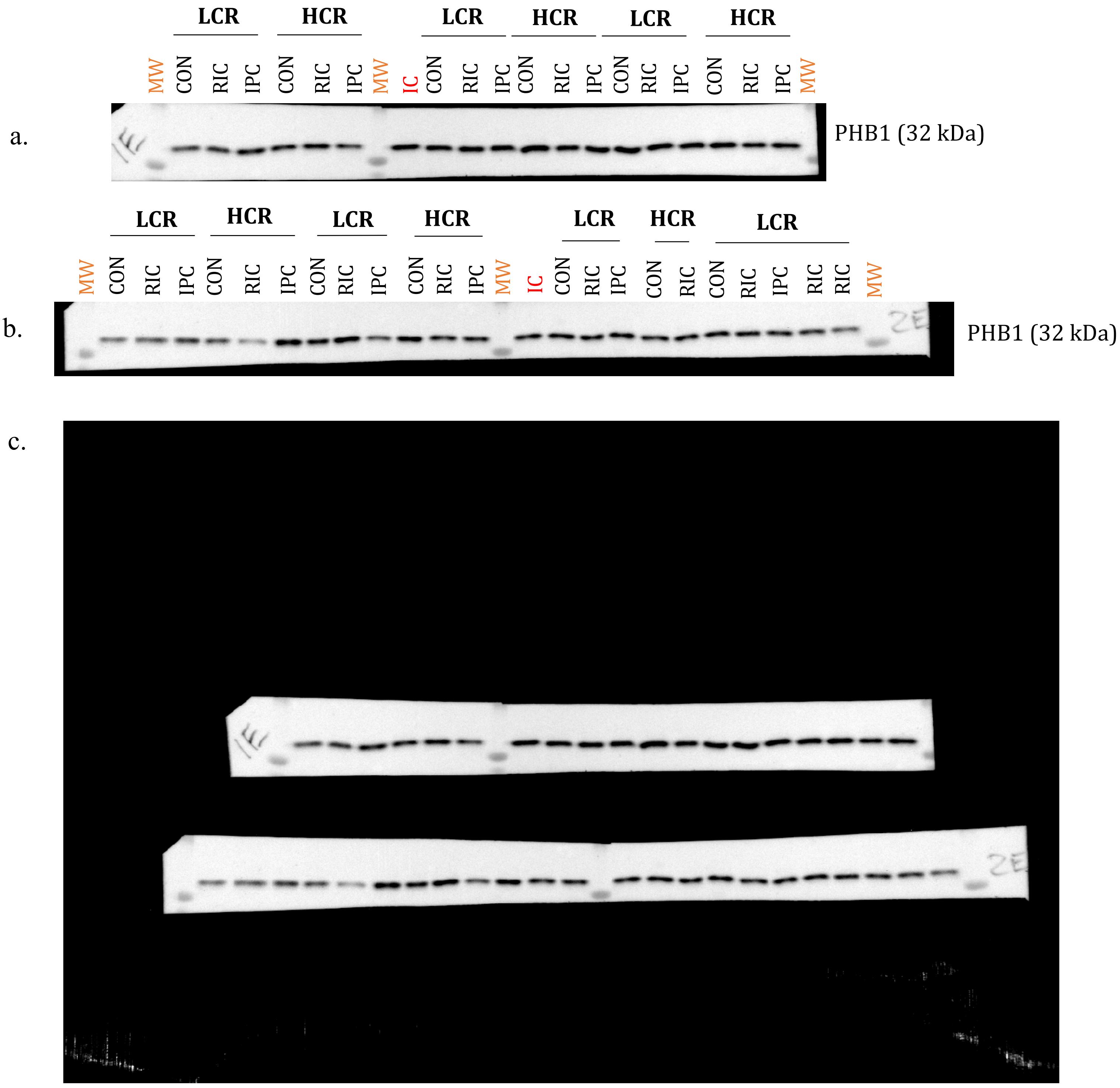

S9. Original blots for PHB1. a. and b shows the bands separately, with makings, c. shows the total uncropped image. Results are shown in figure 3E.
MW: molecular weight marker. IC: internal control.

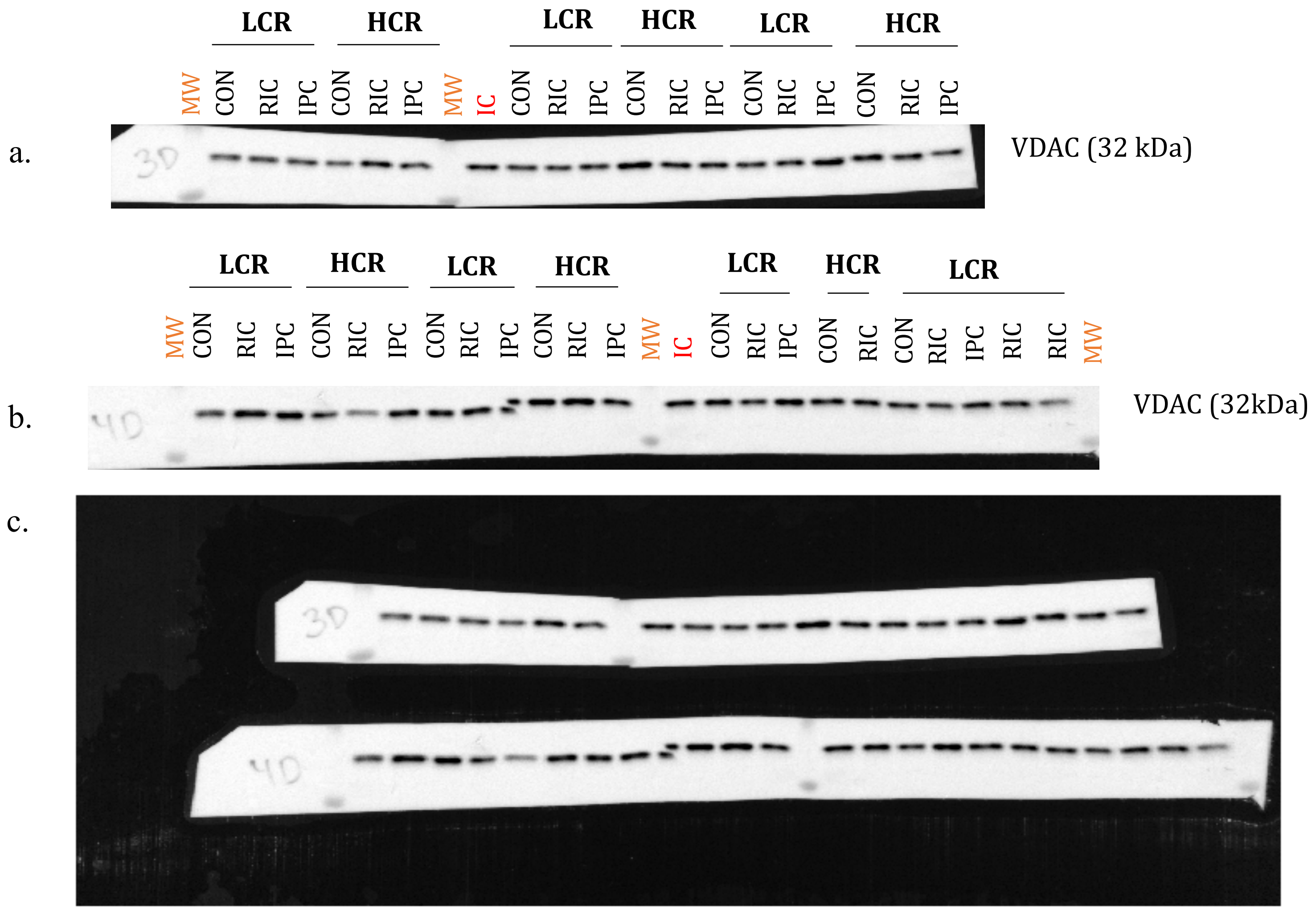

S10. Original blots for total VDAC. a. and b shows the bands separately, with makings, c. shows the total uncropped image. Results are shown in figure 3F.
MW: molecular weight marker. IC: internal control.
* Band 10 (LCR IPC) is in two pieces.

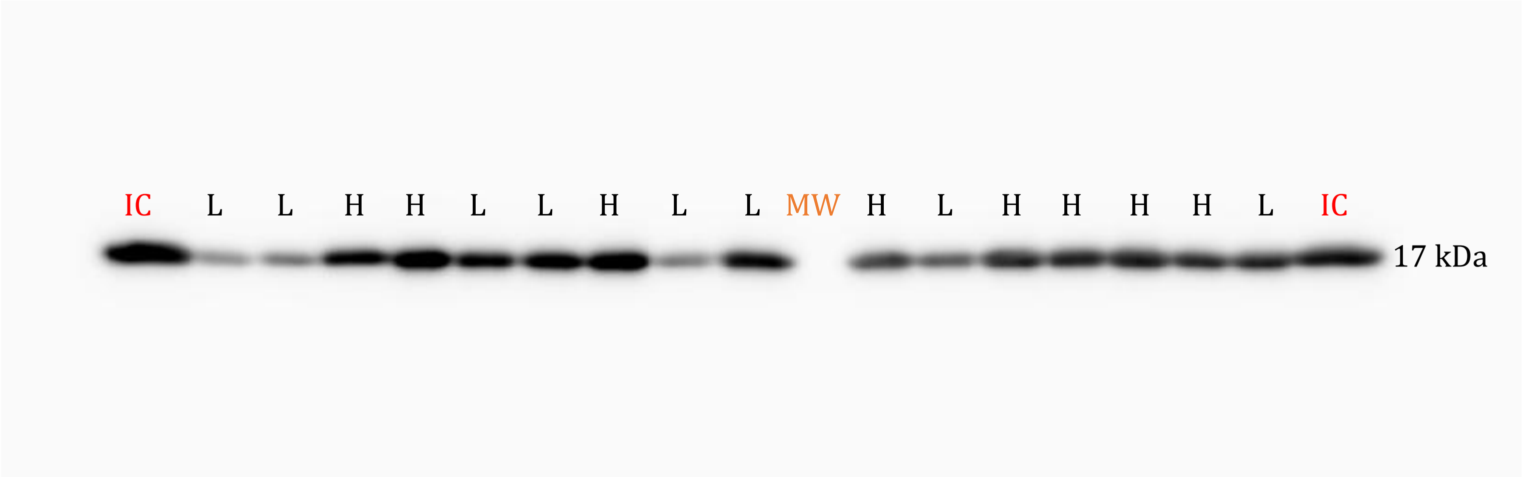

S11. Original blots for COX IV in skeletal muscle. Results are shown in figure 5B.
L: Low capacity runner, H: High capacity runner, MW: molecular weight marker, IC: internal control.

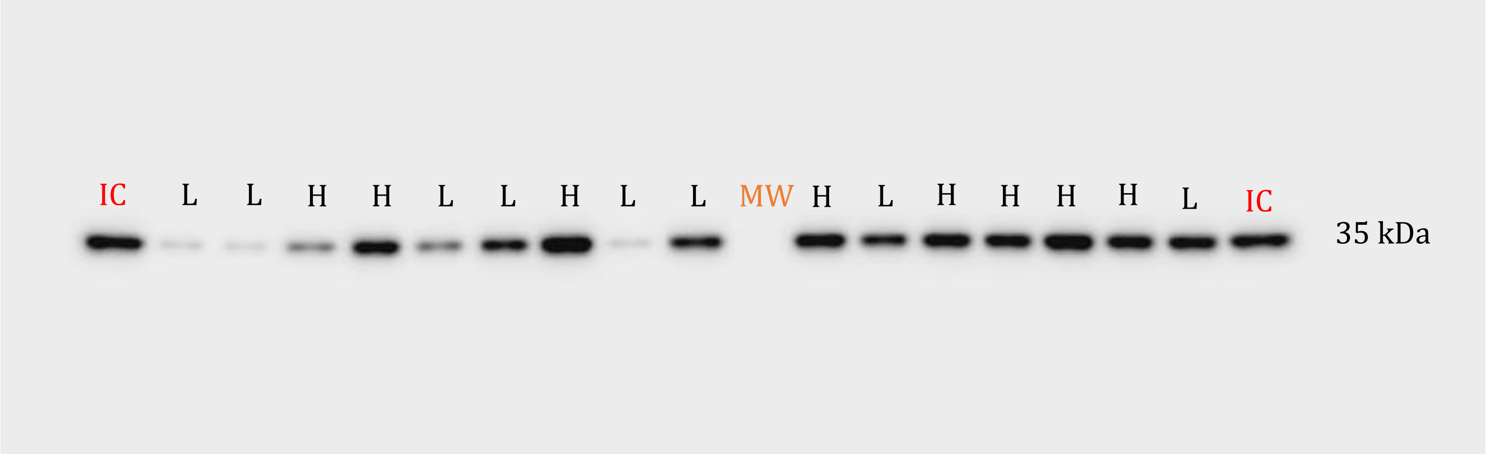

S12. Original blots for β-HAD in skeletal muscle. Results are shown in figure 5B.
L: Low capacity runner, H: High capacity runner, MW: molecular weight marker, IC: internal control.

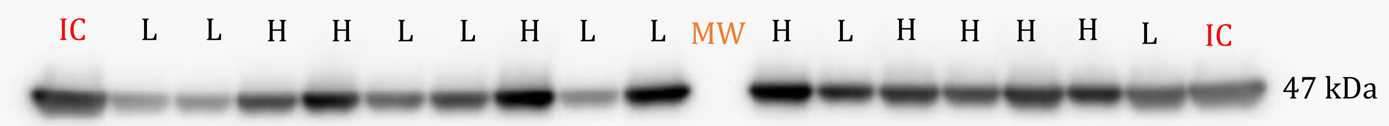

S13. Original blots for CS in skeletal muscle. Results are shown in figure 5B.
L: Low capacity runner, H: High capacity runner, MW: molecular weight marker, IC: internal control.

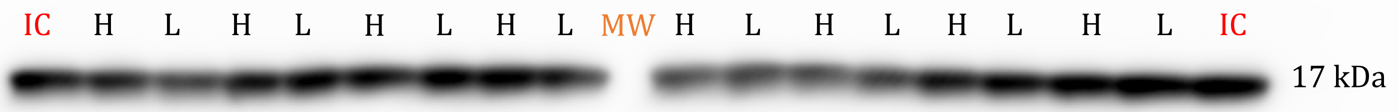

S14. Original blots for COX IV in cardiac muscle. Results are shown in figure 5C.
L: Low capacity runner, H: High capacity runner, MW: molecular weight marker, IC: internal control.

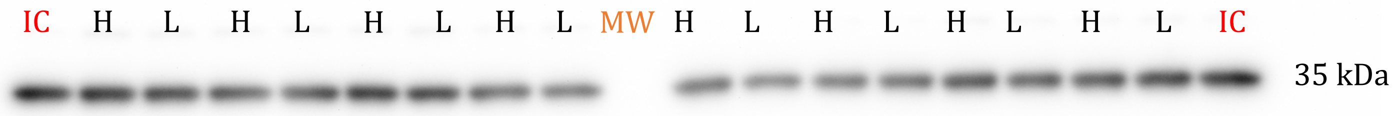

S15. Original blots for β-HAD in cardiac muscle. Results are shown in figure 5C.
L: Low capacity runner, H: High capacity runner, MW: molecular weight marker, IC: internal control.

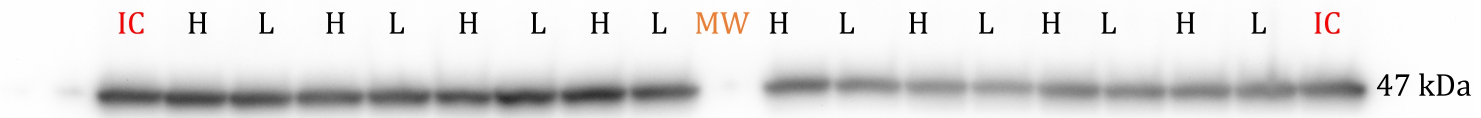

S16. Original blots for CS in cardiac muscle. Results are shown in figure 5C.
L: Low capacity runner, H: High capacity runner, MW: molecular weight marker, IC: internal control.
